# First somatic mutation of E2F1 in a critical DNA binding residue discovered in well-differentiated papillary mesothelioma of the peritoneum

**DOI:** 10.1186/gb-2011-12-9-r96

**Published:** 2011-09-28

**Authors:** Willie Yu, Waraporn Chan-On, Melissa Teo, Choon Kiat Ong, Ioana Cutcutache, George E Allen, Bernice Wong, Swe Swe Myint, Kiat Hon Lim, P Mathijs Voorhoeve, Steve Rozen, Khee Chee Soo, Patrick Tan, Bin Tean Teh

**Affiliations:** 1 NCCS-VARI Translational Research Laboratory, National Cancer Centre Singapore, 11 Hospital Drive, 169610, Singapore; 2 Laboratory of Cancer Therapeutics, Division of Cancer and Stem Cell Biology, Duke-NUS Graduate Medical School, 8 College Road, 169857, Singapore; 3 National University of Singapore Graduate School for Integrative Sciences and Engineering, 28 Medical Drive, 117456, Singapore; 4 Department of Surgical Oncology, National Cancer Centre Singapore, 11 Hospital Drive, 169610, Singapore; 5 Division of Neuroscience and Behavioral Disorders, Duke-NUS Graduate Medical School, 8 College Road, 169857, Singapore; 6 Department of Pathology, Singapore General Hospital - Pathology Building, Outram Road, 169608, Singapore; 7 Laboratory of Molecular Tumor Genetics, Division of Cancer and Stem Cell Biology, Duke-NUS Graduate Medical School, 8 College Road, 169857, Singapore; 8 Department of Biochemistry, Yong Loo Lin School of Medicine, National University of Singapore, 8 Medical Drive - Blk MD7 #02-03, 117597, Singapore; 9 Cancer Science Institute of Singapore, National University of Singapore, 5 Lower Kent Ridge Road, 119074, Singapore; 10 Laboratory of Genomic Oncology, Division of Cancer and Stem Cell Biology, Duke-NUS Graduate Medical School, 8 College Road, 169857, Singapore; 11 Genome Institute of Singapore, 60 Biopolis Street, Genome, #02-01, 138672, Singapore; 12 Laboratory of Cancer Genetics, Van Andel Research Institute, Grand Rapids, Michigan, 49503, USA

## Abstract

**Background:**

Well differentiated papillary mesothelioma of the peritoneum (WDPMP) is a rare variant of epithelial mesothelioma of low malignancy potential, usually found in women with no history of asbestos exposure. In this study, we perform the first exome sequencing of WDPMP.

**Results:**

WDPMP exome sequencing reveals the first somatic mutation of E2F1, R166H, to be identified in human cancer. The location is in the evolutionarily conserved DNA binding domain and computationally predicted to be mutated in the critical contact point between E2F1 and its DNA target. We show that the R166H mutation abrogates E2F1's DNA binding ability and is associated with reduced activation of E2F1 downstream target genes. Mutant E2F1 proteins are also observed in higher quantities when compared with wild-type E2F1 protein levels and the mutant protein's resistance to degradation was found to be the cause of its accumulation within mutant over-expressing cells. Cells over-expressing wild-type E2F1 show decreased proliferation compared to mutant over-expressing cells, but cell proliferation rates of mutant over-expressing cells were comparable to cells over-expressing the empty vector.

**Conclusions:**

The R166H mutation in E2F1 is shown to have a deleterious effect on its DNA binding ability as well as increasing its stability and subsequent accumulation in R166H mutant cells. Based on the results, two compatible theories can be formed: R166H mutation appears to allow for protein over-expression while minimizing the apoptotic consequence and the R166H mutation may behave similarly to SV40 large T antigen, inhibiting tumor suppressive functions of retinoblastoma protein 1.

## Background

Mesothelioma is an uncommon neoplasm that develops from the mesothelium, the protective lining covering a majority of the body's internal organs, and is divided into four subtypes: pleural, peritoneum, pericardium and tunica vaginalis [1]. While malignant peritoneal mesothelioma (MPM) is an aggressive tumor mainly afflicting asbestos-exposed males in the age range of 50 to 60 years old [2], well-differentiated papillary mesothelioma of the peritoneum (WDPMP), a rare subtype of epithelioid mesothelioma [1] with fewer than 60 cases described in the literature [3], is generally considered to be a tumor of low malignant potential found predominately in young women with no definitive exposure to asbestos [3]. While much scientific research has been done on asbestos-related malignant mesothelioma [4-7], the rarity of WDPMP coupled with its good prognosis relegated its research to case reports and reviews by medical oncologists concentrating in the area of diagnosis, prognosis and treatment options.

Second generation sequencing technologies coupled with newly developed whole exome capturing technologies [8] allow for rapid, relatively inexpensive approaches to obtain an overview of large complex genomes by concentrating on the critical coding areas of the genome. Here, we report the first exome sequencing of a WDPMP tumor, its tumor-derived cell line and a matched control sample employing Agilent SureSelect All Exon capturing technology to selectively capture all human exons followed by Illumina massively parallel genomic sequencing. We developed methodology and informatics to obtain a compact graphical view of the exome as well as detailed analysis of single nucleotide variants (SNVs). We demonstrate that while this WDPMP tumor does not exhibit any of the chromosomal aberrations and focal deletions commonly associated with asbestos-related mesothelioma [5], it does exhibit the first reported somatic single nucleotide mutation of *E2F1 *(E2F transcription factor 1) in cancer, with the mutation affecting one of two evolutionarily conserved arginine residues responsible for motif recognition and DNA binding.

## Results

### WDPMP exome sequencing: mutation landscape changes big and small

**Figure 1:**
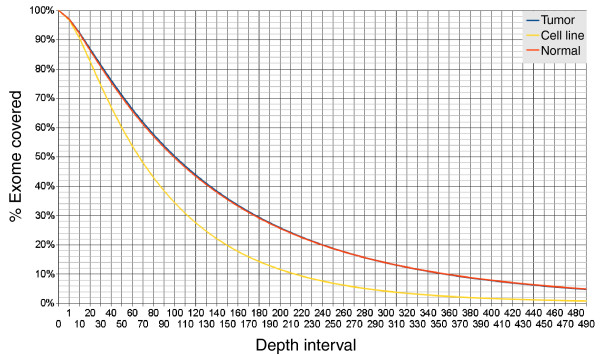
**Cumulative WDPMP exome coverage for the tumor, normal sample and tumor derived cell line**. Cumulative exome coverage curve for the tumor (blue), normal sample (orange) and cell line (yellow) is generated by plotting the percentage of the exome represented by different read depths where read depth is defined as the number of individual 75-bp sequenced reads mapped to a particular exome position. The 'fat tail' of the graph indicates a bias in the capturing technology as small sections of the exome are over-represented.

Exon captured sample libraries comprising DNA from a WDPMP tumor, DNA from the patient's blood, and DNA from a tumor-derived cell line were sequenced using Illumina GAIIx 76-bp paired-end sequencing technology; Table 1 provides a summary of the sequenced exome data for the WDPMP tumor and its matched control sample as well as the tumor-derived cell line; in total, approximately 34 Gbases of sequence data were obtained in which > 92% of the reads successfully mapped back to the hg18 reference genome using the BWA short read aligner [9]. After removal of low quality reads and PCR duplicate reads using SAMtools [10], approximately 24.3 Gbases of sequence data remained. Of the remaining sequence data, approximately 64% (approximately 15.5 Gbases) fell within the exon regions, with the average exome coverage per sample being 152× depth. Figure 1 shows the breakdown of coverage versus sequencing depth; key statistics include that 97% of the exome was covered by at least a single good quality read, approximately 92% of the exome was covered by at least ten good quality reads, and 82 to 86% of the exome was covered by at least 20 reads, indicating that the overall exome capturing and sequencing were successful, generating large amounts of good quality data.

**Table 1:** Summary of overall WDPMP exome sequencing

	Sample ID		
	Tumor	Cell line	Normal
Raw reads	187,023,594	119,030,552	190,772,020
Unalignable reads	14,717,058	3,778,934	14,190,612
Aligned reads	172,306,536	115,251,618	176,581,408
Percentage alignable	92.13%	96.83%	92.56%
Reads passing filter	129,919,859	92,512,679	137,134,828
Percentage remaining after filter	69.47%	77.72%	71.88%
Number of PCR duplicates	8,498,978	15,903,654	6,070,718
Percentage PCR duplicates	6.54%	17.19%	4.43%
Reads within exome	80,042,870	49,312,008	80,869,734
Percentage reads overall in exome	61.61%	53.30%	58.97%
Percentage exome covered at 1×	97.10%	96.80%	97.00%
Percentage exome covered at 20×	86.80%	82.40%	86.30%

**Figure 2:**
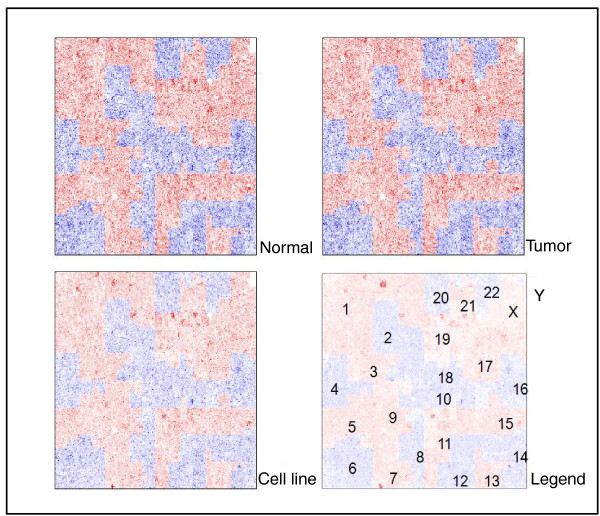
**Compact representation of the WDPMP exome using Hilbert plots**. Instead of linearly plotting the sequencing depth versus the exome DNA string, HilbertVis [11] computationally wraps the DNA string in a fractal manner onto a two-dimensional grid of pre-determined size and represents the coverage depth via a heat map similar to gene expression data. Red and blue color heat mapping is used to demarcate the borders of each chromosome.

A novel way to visualize large copy number changes using exome sequencing data is the use of HilbertVis [11], an R statistical package, to plot exome sequencing depth versus chromosomal position in a compact graphical manner. Copy number changes, if present, will reveal themselves through color intensity changes in regions of the plot where copy number change occurs when comparing between tumor/cell line versus normal samples. Figure 2 shows the Hilbert plots of the sequenced tumor, tumor-derived cell line and normal blood sample exomes, revealing some systemic capturing biases but no deletion/amplification events, with particular attention paid to known somatic deletions of 3p21, 9p13~21 and 22q associated with loss of *RASSF1A *(RAS association family 1A), *CDKN2A *(cyclin-dependent kinase inhibitor 2A) and *NF2 *(neurofibromin 2) genes, respectively, in malignant mesothelioma [12]. Sequencing depth was also adequate for the regions of exon capture for these genes (Additional file 1), indicating these genes were truly not somatically mutated and that any lack of detected mutations was not due to a lack of coverage.

Since the Hilbert plots showed no gross anomalies, we turned our attention to mining the exome data for somatic single nucleotide mutations. The SNV discovery pipeline, described in the Materials and methods section, was performed using the Genome Analyzer Toolkit [13] for the tumor, normal and cell line exomes. Filtering was set to accept candidate SNVs with a quality/depth score > 3 and were present in both the tumor and cell line but not in the normal sample. Nineteen potential somatic mutations remain and these were validated using Sanger sequencing (Additional file 2); *E2F1*, *PPFIBP2 *(liprin beta 2) and *TRAF7 *(TNF receptor-associated factor 7) were validated to contain true somatic mutations (Additional file 3).

### The E2F1 R166H mutation affects a critical DNA binding residue

**Figure 3:**
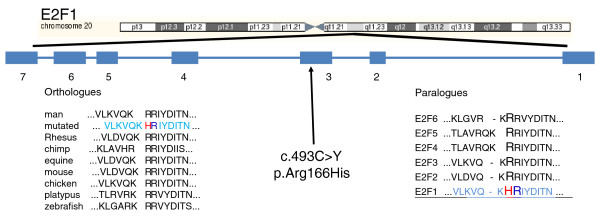
**Location and conservation analysis of E2F1 R166H**. *E2F1 *genomic location, exon location of c.493 c > Y mutation and results of *E2F1 *mutation validation and conservation analysis. Top: the chromosomal location of *E2F1 *and the location of its exons. The exon numbering indicates *E2F1 *is located on the reverse strand and the c.493C > Y mutation is location on exon 3, which translates to a p.Arg166His residue mutation. E2F1 orthologue conservation analysis was performed using the SNP Analysis function of SNPs3D [15] with the E2F1 mutated protein sequence shown in light blue (bottom left). The arginine-arginine conservation across diverse species is shown with the histidine mutation highlighted in red and its arginine partner highlighted in blue. E2F1 paralogue conservation analysis was performed using CLUSTALW [14] at default settings (bottom right). The E2F1 mutated sequence is shown in light blue and underlined with the histidine mutation shown in red and its partner arginine shown in blue. Again the arginine-arginine conservation across the E2F family is clearly shown.

The E2F1 R166H somatic mutation is of particular interest as there is no reported mutation of the *E2F1 *gene in cancer. Figure 3 (top) shows the genomic location of *E2F1 *as well as the specific location of the mutation. Sanger sequencing around the mutated nucleotide of the tumor, cell line and normal sample revealed the mutation to be heterozygous (Additional file 3). A check of UniProt for E2F1 [UniProtKB: Q01094] showed the mutation to be located in the DNA binding domain of the protein. To study the evolutionary conservation of the R166 residue, a CLUSTALW [14] analysis was performed on paralogues of the human E2F family and SNP analysis, using SNPs3D [15], was performed across orthologues of E2F1. Figure 3 (bottom) shows the results of the paralogue and orthologue conservation analyses; the conclusion drawn is that the R166 residue is conserved in evolution and has never been observed to be mutated.

**Figure 4:**
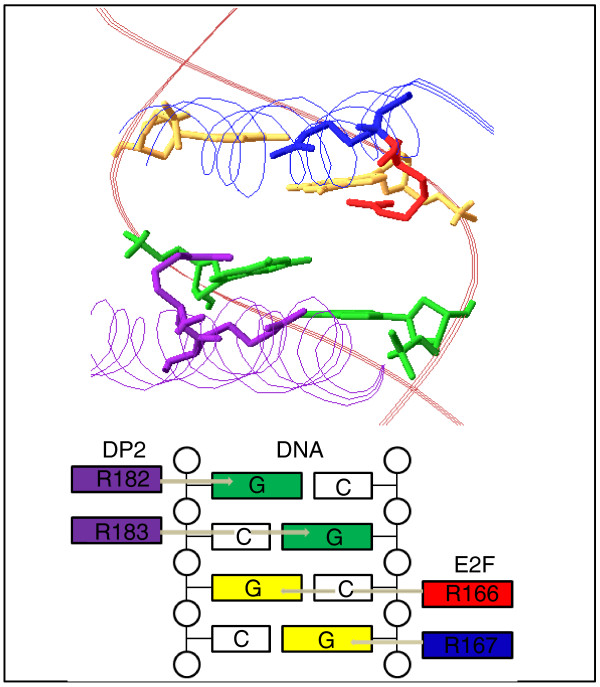
**Visualization of the p.Arg166His mutation in E2F1**. Top: the E2F4 crystal structure [PDB:1CF7] showing the location of the p.Arg166His mutation. The brown double helix is the DNA binding motif with green colored guanine nucleotides representing binding targets of Arg182 and Arg183 of the DP2 protein and yellow colored guanine nucleotides representing binding targets of Arg166 and Arg165 of the E2F protein. The blue ribbon represents the DNA binding region of E2F with the Arg166 mutation in red and Arg165 in blue, while the purple ribbon represents the DNA binding region of DP2 with Arg182 and Arg183 in purple. Bottom: a schematic showing binding of E2F residues to DNA binding site nucleotides.

Since there is no E2F1 crystal structure containing the R166 residue, the X-ray crystal structure of E2F4-DP [PDB:1CF7] was used to determine the location of the mutation and its role in DNA binding using the Swiss-PDB viewer [16]. The E2F4 DNA binding structure was used as an adequate representation of its E2F1 counterpart due to the conserved status of the R165-R166 residues across the E2F paralogues (Figure 3, bottom right) as well as the affected residue being a part of the winged-helix DNA-binding motif observed across the whole E2F family of transcription factors [17]. The arginine residues of E2F4 and its dimerization partner DP are responsible for DNA binding (Figure 4, top) and the analysis clearly shows R166 as one of four arginine residues contacting the DNA target (Figure 4, bottom).

**Figure 5:**
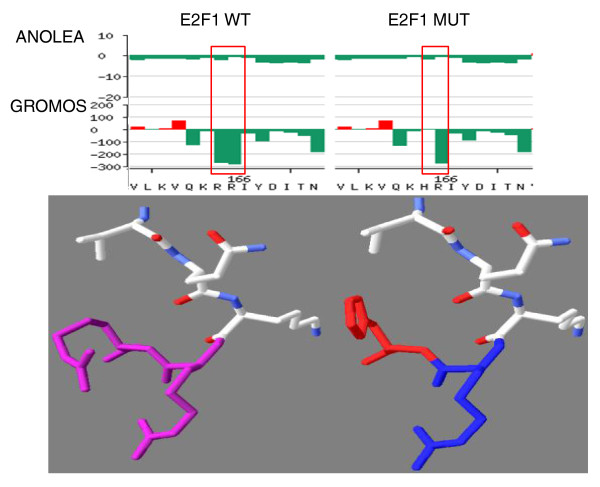
**Homology modeling of wild-type and mutant E2F1 around the R166 residue**. Homology modeling of the E2F1 DNA binding domain using SWISS-MODEL [18]. Top: ANOLEA (Atomic Non-Local Environment Assessment) [19] and GROMOS (Groningen Molecular Simulation) [20] were used by SWISS-MODEL to assess the quality of the model structure of the E2F1 wild-type and E2F1 R166H mutant DNA binding domain. The y-axis represents the energy for each amino acid of the protein, with negative energy values (in green) representing a favorable energy environment and positive energy values (in red) representing unfavorable energy environments. Bottom: the predicted three-dimensional structure of residues VQK(R/H)R with the wild-type arginine-arginine residues shown in purple (bottom left), the mutated histidine residue shown in red and its arginine neighbor shown in blue (bottom right). The side chain of the histidine mutation is clearly predicted to be oriented approximately 90 degrees counterclockwise compared to the side chains of its wild-type arginine counterpart.

Since the crystal structure for the DNA binding domain of E2F4 was available, computational modeling of the mutation was amenable to homology modeling using SWISS-MODEL [18]. Figure 5 (top) shows the modeling of the E2F1 mutant and wild-type DNA binding domain; Calculation of individual residue energy using ANOLEA (Atomic Non-Local Environment Assessment) [19] and GROMOS (Groningen Molecular Simulation) [20] indicated that the mutant histidine's predicted position and conformation were still favorable as indicated by the negative energy value (Figure 5, bottom). While there is a difference in the size and charge between the mutant histidine and wild-type arginine residue coupled with a conformational shift at the mutated position, the overall three-dimensional structure of the domain appears minimally affected by the mutation. Even though the effect of the mutation on DNA binding is computationally inconclusive, these results did pinpoint the structural location and functional importance of the R166 residue, thus pointing the way for the functional experiments reported below.

### The R166H mutation is detrimental to E2F1's DNA binding ability and negatively affects downstream target gene expression

**Figure 6:**
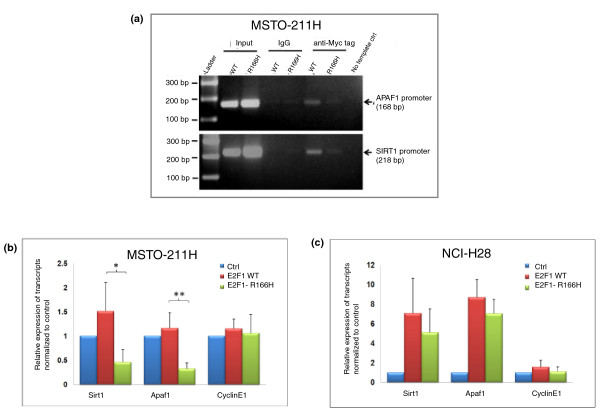
**Mutation of R166 in E2F1 affects its efficiency of binding to promoter targets**. **(a) **ChIP assay on MSTO-211H cells transiently transfected with E2F1 wild type (WT) or E2F1-R166H (R166H) for 48 hours using anti-Myc antibody. The amplification levels of the *APAF1 *(top) and *SIRT1 *(bottom) promoters were determined by PCR. Anti-IgG antibody was used as negative control. **(b, c) **Expression levels of E2F1 targets - *SIRT1*, *APAF1*, and *CCNE1 *- in MSTO-211H and NCI-H28 cells that were transfected with the indicated plasmids. Each bar represents mean ± standard deviation (*n *= 3; *P < 0.05, **P < 0.01). Ctrl, empty vector.

In order to conclusively show the effect of the R166H mutation on DNA binding, chromatin immunoprecipitation (ChIP) assays targeting the *SIRT1 *(sirtuin 1) and *APAF1 *(apoptotic peptidase activating factor 1) promoters using the MSTO-211H cell line over-expressing E2F1 (wild type and mutant) were performed. The mutant E2F1 (Figure 6a, lane 7) showed significantly decreased levels of *APAF1 *(top) and *SIRT1 *promoter DNA binding (bottom) when compared with wild-type E2F1 (Figure 6a, lane 6), although the amount of input DNA for the E2F1 mutant was greater than for the E2F1 wild type (Figure 6a, lanes 2 and 3, respectively). The ChIP result indicates that the R166H mutation has a detrimental effect on E2F1's DNA binding ability.

To show that the R166H mutant's reduced DNA binding affinity affected the expression of E2F1 target genes, the expression of *SIRT1*, *APAF1 *and *CCNE1 *(cyclin E1) was examined by real-time PCR in MSTO-211H and NCI-H28 cell lines that were transfected with the E2F1 mutant or wild type. Interestingly, over-expression of the E2F1 R166H mutant (E2F1-R166H) did not up-regulate expression of *SIRT1 *and *APAF1 *as high as over-expression of wild-type E2F1 in both cell lines (Figure 6b, c). In particular, levels of *SIRT1 *and *APAF1 *expression in MSTO-211H cells observed with E2F1-R166H were significantly lower than those with the E2F1 wild type (*P *= 0.032 for *SIRT1 *and *P *= 0.005 for *APAF1*). However, the expression of cyclin E1, a well known target of E2F1 [21], was minimally affected in the over-expression context, which may be indicative of a compensatory effect by other members of the E2F family.

### Cells over-expressing E2F1-R166H show massive protein accumulation and increased protein stability

**Figure 7:**
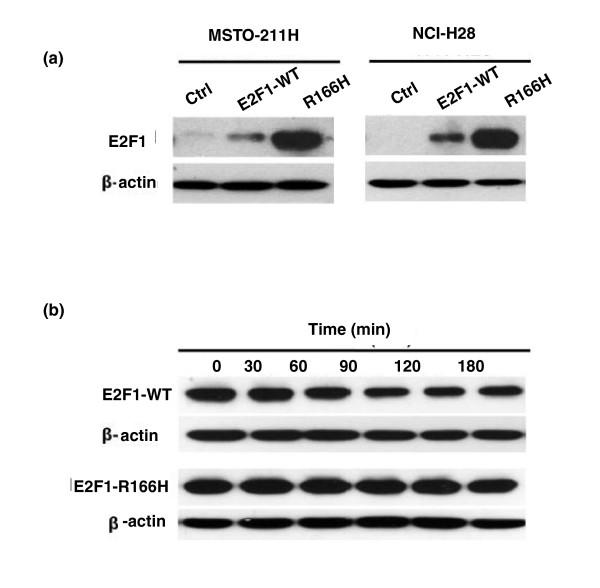
**Accumulation of mutant E2F1 protein in cells due to increased stability of E2F1-R166H**. **(a) **E2F1 protein levels detected by anti-E2F1 antibody (KH95) 48 hours after transfection. WT, wild type. **(b) **Degradation assay performed in MSTO-211H cells over-expressing E2F1 treated with 25 μg/ml cycloheximide. Levels of E2F1 protein were monitored every 30 minutes for 3 hours using anti-E2F1 antibody.

To study cellular phenotypes that might be affected by the R166H mutation, we initially over-expressed the mutant and wild type in the MSTO-211H and NCI-H28 cell lines. Surprisingly, an obvious difference in E2F1 protein levels between wild type and mutant was observed in both cell lines as determined by western blot (Figure 7a). In order to ensure the protein differences were not due to differences in transfection efficiency, the two cell lines were co-transfected with E2F1 and enhanced green fluorescent protein (EGFP) vectors simultaneously with protein lysate obtained at 48 hours after transfection for western blot analysis. Clearly, the levels of expression of the E2F1 wild type and mutant were similar when normalized to EGFP levels (Additional file 4), indicating that the transfection efficiency of E2F1-R166H is not different from that of the wild type. This suggests that the large increase in the level of the mutant E2F1 protein might be caused by other mechanisms, such as increased protein stability.

To monitor E2F1 protein stability, we over-expressed the E2F1 wild type and mutant in MSTO-211H cells before treating the cells with 25 μg/ml cyclohexamide to block new protein synthesis in half hour intervals. As shown in Figure 6b, the protein levels of the E2F1 mutant remained almost constant throughout the 3-hour period of the experiment while those of the wild type decreased in a time-dependent manner. This result suggests that the mutant protein is more stable and resistant to degradation than the wild type and the increased stability of E2F1-R166H is the cause of its accumulation within cells over-expressing it.

### Over-expression of E2F1-R166H does not adversely affect cell proliferation

**Figure 8:**
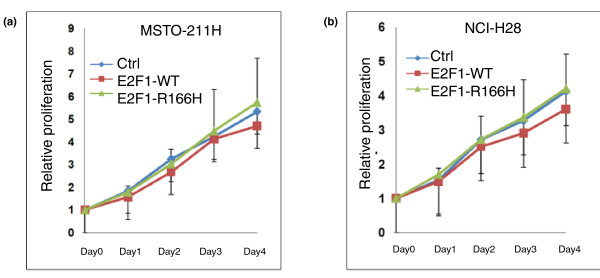
**Over expression of the E2F1 R166H mutant in two mesothelial cell lines**. (a, b) Proliferation assay after over-expressing the E2F1wild type (E2F1-WT) or mutant (E2F1-R166H) or empty vector (Ctrl) in MSTO-211H and NCI-H28 cells. Cells were transfected with the indicated plasmids for 48 hours. Data are means ± standard deviation (*n *= 3).

Since the R166H mutant is demonstrated to have exceptional stability and accumulates greatly in cells over-expressing it, it would be instructive to observe what effect, if any, it has on cell proliferation. A proliferation assay was performed on the transiently transfected cell lines. The results showed that high expression of the E2F1 wild type slightly decreased the growth rate of cells whereas high expression of the mutant resulted in a slightly better growth rate (Figure 8a, b). Although E2F1-R166H does not show a significant effect on regulating cell proliferation, it is possible that the mutation is advantageous to cancer cells as it does not inhibit cell growth when the mutant is highly expressed in cells.

## Discussion

For this study we have performed the first exome sequencing of a matched pair of WDPMP samples along with a cell line derived from the tumor. Analysis of the exomes revealed none of the chromosomal aberrations or focal gene deletions commonly associated with asbestos-related malignant mesothelioma. We were able to verify somatic mutations in *PPFIBP2*, *TRAF7 *and *E2F1*.

TRAF7 is an E3 ubiquitin ligase [21] shown to be involved in MEKK3 (mitogen-activated protein kinase kinase kinase 3) signaling and apoptosis [22]. The Y621D mutation in TRAF7 occurs in the WD40 repeat domain, which has been shown to be involved in MEKK3-induced activator protein 1 (AP1) activation [22]. Since AP1 in turn controls a large number of cellular processes involved in differentiation, proliferation and apoptosis [23], this mutation in TRAF7's WD40 repeat domain may de-regulate MEKK3's control over AP1 activation, which may contribute to WDPMP transformation.

PPFIBP2 is a member of the LAR protein-tyrosine-phosphatase-interacting protein (liprin) family [24]. While no functional studies on PPFIBP2 have been published, it was reported as a potential biomarker for endometrial carcinomas [25]. However, the Q791H mutation in PPFIBP2 is predicted by Polyphen to be benign and the Catalogue of Somatic Mutations in Cancer (COSMIC) did not show this particular mutation to occur in other cancers; thus, this mutation is likely to be of a passenger variety.

Of particular interest is the E2F1 mutation as no reported somatic mutation has ever been reported for this protein despite its critical roles in cell cycle control [26], apoptosis [27] and DNA repair [28]. Using various bioinformatics tools, this mutation was identified to mutate an arginine residue into a histidine residue, thus altering a critical evolutionarily conserved DNA contact point responsible for DNA binding and motif recognition.

Since computational modeling is sufficient to pinpoint the mutation's structural location but is inconclusive in showing the mutation's functional effect on DNA binding, we performed a ChIP assay that showed the R166H mutation abrogates E2F1 DNA binding. Analysis of expression of selected E2F1 target genes in an over-expression system showed the inability of the E2F1 mutant to adequately up-regulate the expression of *SIRT1 *and *APAF1 *when compared with the E2F1 wild type. Of interest is the lack of changes in the expression of the gene encoding cyclin E1, a known target of E2F1 and an important component in starting the S phase of the cell cycle. A possible explanation for this is the functional redundancy of the E2F family to ensure the cell's replication machinery is operational - for example, mice studies have shown that *E2F1*-/- mice can be grown to maturity [29,30].

Our study has also shown that the R166H mutant is much more stable than its wild-type counterpart, enabling massive accumulation within the cell. A previous study showed that over-expression of E2F1 results in induction of apoptosis [31], which is in line with our observation of a drop in proliferation when cells were over-expressing wild-type E2F1; curiously, over-expression of the mutant E2F1 protein did not lead to any noticeable effect on cellular proliferation even though mutant protein levels were many fold higher than those of the wild type in equivalent transfection conditions. One explanation for this phenomenon is that inactivation of E2F1 decreases apoptosis and its abrogated cell cycle role is compensated for by other members of its family. *E2F1*-/- mice can grow to maturity and reproduce normally but display a predisposition to develop various cancers [30], indicating the greater importance of the tumor suppressive function of E2F1 compared to its cell cycle gene activation function.

An alternative but not mutually exclusive explanation is that stable and numerous E2F1-R166H proteins behave functionally like SV40 large T antigens, serving as competitive inhibitors by taking up the lion's share of the binding capacity of retinoblastoma protein 1 (Rb), resulting in unbound wild-type E2F1, which drives the cell cycle. While the R166H mutation cripples E2F1's DNA binding ability, its other interaction domains, including the Rb interaction domain, are still active. The mutant's stability and large quantities will favor its preferential binding to Rb due to its sheer numbers and the heterozygous nature of the mutation in the WDPMP tumor would ensure active copies of wild-type E2F1 were present to drive the cell cycle. This theory is supported by two studies: Cress *et al. *[32] created an E2F1-E132 mutant that is artificially mutated in position 132 within E2F1's DNA binding domain and that was demonstrated to have lost its DNA binding capacity, like our R166H mutant; Halaban *et al. *[33] demonstrated that expression of the E2F1-E132 mutant can induce a partially transformed phenotype by conferring growth factor-independent cell cycle progression in mice melanocytes. One possible reason why the proliferation of cells over-expressing the E2F1 mutant was not greater than that of control cells is that both mesothelial cell lines used in this study already have a homozygous deletion of the *CDKN2A *gene resulting in p16 null cells. A key part of the G1/S checkpoint of the cell cycle is p16 deactivation of cyclin-dependent kinase 6, which keeps Rb hypophosphoyylated, thus keeping E2F1 sequestered [34]. A p16 null cell has already lost its G1/S checkpoint control; thus, introducing another mutation that will cause the same checkpoint loss will not cause noticeable growth differences.

Given that WDPMP is a rare sub-type of mesothelioma, it is of interest to extrapolate E2F1's role to the more prevalent MPM. Given that *CDKN2A *homozygous deletion is prevalent in MPM, with up to 72% of tumors affected [35], the G1/S checkpoint is already broken in *CDKN2A *deleted tumors; thus, in terms of proliferation it is unlikely that an additional E2F1 R166H mutation will be useful as the mutation will be redundant in this context. On the other hand, E2F1 also plays an important role in the activation of apoptosis pathways [27], and the R166H mutation, with its abrogated DNA binding, may contribute to the survival of the cancer cell harboring this mutation. It would be worth checking the remaining 28% of MPMs without *CDKN2A *deletion for possible mutations in *E2F1 *and other related genes. It is interesting to note that BAP1 (BRCA1 associated protein-1), a nuclear deubiquitinase affecting E2F and Polycomb target genes, was recently shown to be inactivated by somatic mutations in 23% of MPMs [36], suggesting that the genes within the E2F pathways might play an important role in mesothelioma in general.

## Conclusions

We have performed the first exome sequencing of a WDPMP tumor and a matched control sample and a tumor-derived cell line and discovered the first somatic mutation of E2F1, R166H. This mutation is found to be the critical DNA contact point in the protein's DNA binding domain responsible for gene activation and motif recognition. Experiments confirmed that the mutation abrogates DNA binding and renders the mutated protein unable to adequately up-regulate its target genes. Large accumulation of the mutant protein is observed in over-expression studies and this is due to a great increase in protein stability as shown by a cyclohexamide chase assay. Overall, two compatible theories can explain the observed results: first, E2F1-R166H decreases apoptosis and its abrogated cell cycle role is compensated for by other members of its family; and second, heterozygous E2F1-R166H behaves like SV-40 large T antigen, interfering with the tumor suppressive role of Rb and allowing its wild-type counterpart to drive cell division.

## Materials and methods

### Patient materials

Tumor and blood samples were collected from a 41-year-old Chinese female who was diagnosed with WDPMP after a laparoscopic biopsy of the omental nodules that were found during a computerized tomography scan. The patient underwent cytoreductive surgery and hyperthermic infusion of intraperitoneal chemotherapy. She completed 5 days of early post-operative intraperitoneal chemotherapy whilst hospitalized, and recovered uneventfully without any complications. She was discharged on post-operative day 15 and remains disease-free at 8 months after her surgery. Informed consent for tissue collection was obtained from the patient by SingHealth Tissue Repository (approved reference number 10-MES-197) and this study was approved by the SingHealth Centralised Institutional Review Board (CIRB reference number 2010-282-B).

### Cell line establishment

Fresh tumor sections were first minced into a paste using surgical scissors in a sterile petri dish and the minced section was transferred to a 50-ml falcon conical tube along with 10 ml of 0.1% collagenase (C5138; Sigma, St. Louis, MO, USA) and incubated for 1 hour at 37°C. RPMI1640 (40 ml) was then added to the tube and spun for 5 minutes at 500 g after which the supernatant was removed and the process repeated until the pellet had a white color. The pellet was re-suspended with 14 ml of RPMI1640 containing 10% fetal bovine serum and antibiotics and seeded onto a T-75 flask. The flask was incubated for 24 hours at 37°C in a 5% CO_2 _environment before being checked under the microscope for cell attachment to the flask surface and the cells were passaged every 3 days.

### Extraction of DNA from patient samples and cell lines

For sample DNA extraction, approximately 15 to 20 mg of frozen tissue was measured out and the sample pulverized into a fine powder using a mortar and pestle; the powdered sample was then added to a 15-ml falcon tube containing 2 ml Master mix containing 4 μl of Rnase A, 100 μl of QIAGEN (Valencia, CA, USA) protease and 2 μl of Buffer G2 and mixed thoroughly. The mixture was incubated in a 50°C incubator for 24 hours then spun at maximum speed for 25 minutes before the supernatant was extracted.

DNA was then extracted from the supernatant using QIAGEN's Blood and Cell Culture Mini kit according to the manufacturer's instructions. In brief, the supernatant was loaded into the kit-supplied column (Genomic-Tip 20/G) and the flow-through was discarded. The column was then washed and DNA eluted into a falcon tube and isopropanol added to precipitate the DNA. The tube was then spun at maximum speed for 15 minutes before washing twice with 70% ethanol. The ethanol was discarded and the remaining DNA pellet re-suspended in TE buffer.

### Exome capture and paired-end sequencing

Sample exomes were captured using Agilent SureSelect Human All Exon Kit v1.01 designed to encompass 37.8 Mb of the human exon coding region. DNA (3 μg) from the WDPMP tumor, matched blood sample and the tumor-derived cell line were sheared, end-repaired and ligated with paired-end adaptors before hybridizing with biotinylated RNA library baits for 24 hours at 65°C. The DNA-bait RNA fragments were captured using streptavidin-coated magnetic beads and the captured fragments were RNA digested, with the remaining DNA fragments PCR amplified to generate the exon captured sequencing library.

A 15 picomolar concentration of the exome library was used in cluster generation in accordance with Illumina's v3 paired-end cluster-generation protocol. The cluster-generated flow cell was then loaded into the GAIIx sequencer to generate the 76 bp of the first read. After first read completion, the paired end module of GAIIx was used to regenerate the clusters within the flow cell for another 76 bp sequencing of the second read. All raw sequencing data generated are available at the NCBI Sequence Read Archive [37] [SRA:SRP007386].

### Sequence mapping and filtering criteria

Illumina paired-end reads were first converted from Illumina quality scores to Sanger quality scores using the converter module of MAQ before paired-end read alignment to the NCBI hg18 build 36.1 reference genome using the short read aligner BWA (Burrows-Wheeler Aligner) [9] with default options. The aligned output from BWA was processed by SAMtools [10] in the following manner. The BWA output was first converted into a compressed BAM format before the aligned sequences were sorted according to chromosomal coordinates. The sorted sequences were then subjected to SAMtools' PCR duplicate removal module to discard sequence pairs with identical outer chromosomal coordinates. Because each sample was sequenced in duplicate, the resulting BAM files representing the duplicate lanes were merged into a single BAM file before the quality filtering step. Quality filtering involved selecting sequences that were uniquely aligned with the reference genome, had less than or equal to four mismatches to the reference genome and had a mapping quality score of at least one. The output result of this filter formed the core sequence file for further downstream analysis.

### Generation of exome Hilbert plots

Using the core sequence file described above, we first discarded all intronic bases in the following manner. First, conversion was performed on Agilent's SureSelect exon coordinates file from BED format into space-delimited format specifying the chromosomal location of every exon base. SAMtools' pileup command, using the space-delimited exon coordinate file as a parameter, was used to exclusively output only bases belonging to the exome. Since the pileup command was coded to output only bases with non-zero depth to conserve storage, a quick R script was used to insert in the exome bases that are of zero depth into the initial exome pileup output. This final pileup contains every nucleotide of the exome and its associate sequencing depth sorted by chromosomal coordinates. For the visualization of the entire exome, we used the statistical program R, and in particular HilbertVis, a compact graphical representation of linear data packages [11]. Instead of linearly plotting the sequencing depth versus the exome DNA string, a Hilbert plot computationally wraps the DNA string in a fractal manner onto a two-dimensional grid of pre-determined size and represents the coverage depth via a heat map similar to gene expression data. Red and blue color heat mapping is used to demarcate the borders of each chromosome.

### Single nucleotide variant discovery

Additional file 5 shows the SNV discovery pipeline. Aligned reads were processed using Genome Analyzer Toolkit [13]. Reads containing microindels were first locally re-aligned to obtain more accurate quality scores and alignments then quality filtered before consensus calling was performed to obtain the raw SNVs. These raw SNVs were subjected to further quality filtering before being compared against dbSNP130 and 1000 Genomes databases where common SNPs present in the exome were discarded; from this pool of remaining SNVs, only non-synonymous variations occurring in exons or splice sites were retained. This pipeline was performed for tumor, normal sample and cell line exomes and only SNVs that had a quality/depth score > 3 and were present in both the tumor and cell line and not in the normal sample were retained; SNVs in this final pool were considered to be candidate somatic mutations.

### Sanger sequencing validation

Primers for sequencing validation were designed using Primer3 [38]. Purified PCR products were sequenced in forward and reverse directions using the ABI PRISM BigDye Terminator Cycle Sequencing Ready Reaction kit (version 3) and an ABI PRISM 3730 Genetic Analyzer (Applied Biosystems, Foster City, CA, USA). Chromatograms were analyzed by SeqScape V2.5 and by manual review. The validation PCR primers were (where F and R stand for forward and reverse, respectively): E2F1_F, 5' GCAGCCACAGTGGGTATTACT 3'; E2F1_R, 5' GGGGAGAAGTCACGCTATGA 3'; TRAF7_F, 5' GCCTTGCTCAGTGTCTTTGA 3'; TRAF7_R, 5' CATGTTGTCCATACTCCAGACC 3'; PPFIBP2_F, 5' CCCTCGAGCCATTTGTATTT 3'; PPFIBP2_R, 5' CCACAGCAGAAGCTGAAAGA 3'.

### Protein visualization and homology modeling

Protein modeling of the mutated and wild-type DNA binding domain of E2F1 was done using the automated mode of SWISS-MODEL [18], a web-based fully automated protein structure homology-modeling server. The basic input requirement from the user is the protein sequence of interest or its UniProt AC code (if available). Swiss-PDBviewer [16] provides an interface allowing users to visualize and manipulate multiple proteins simultaneously. Structures generated by SWISS-MODEL or experimentally determined structures archived at the RCSB Protein Data Bank [39] can be downloaded in a compact.pdb format that serves as the input source for this viewer.

### Mesothelioma cell lines and mutant plasmid generation

The mesothelioma cell lines MSTO-211H and NCI-H28 (ATCC catalogue number CRL2081 and CRL5820, respectively) were cultured in RPMI-1640 supplemented with 10% fetal bovine serum (v/v). Total RNA extracted from the heterozygous E2F1 mutated mesothelioma sample was used for cDNA synthesis using an iScrip cDNA Synthesis Kit (Bio-Rad, Hercules, CA, USA). Full-length wild-type and mutant *E2F1 *were amplified using iProof DNA polymerase (Bio-Rad) and *E2F1 *primers. The primer sequences were: E2F1-ORF-F, 5'-AGTTAAGCTTGACCATGGCCTTGGCCGGGG-3'; E2F1-ORF-R, 5'-AGAATTCCAGAAATCCAGGGGGGTGAGGT-3'. The PCR products were subsequently cloned into pcDNA6/*myc*-His B (Invitrogen, Carlsbad, CA, USA) using HindIII and EcoRI. Plasmids expressing wild-type E2F1 (pcDNA6-E2F1) or mutant E2F1 (pcDNA6-E2F1/R166H) were validated by dideoxy terminator sequencing. pcDNA3-EGFP was constructed as described previously [40].

### Chromatin immunoprecipitation

ChIP was carried out in MSTO-211H cells transiently transfected with wild-type E2F1 and E2F1-R166H for 48 hours. Transiently transfected cells were cross-linked with 1% formaldehyde. Chromatin solution pre-cleared with protein G sepharose 4 fast flow (GE Healthcare Life Sciences, Piscataway, NJ, USA) was used for immunoprecipitation with anti-Myc tag antibody (ab9132; Abcam, Boston, MA, USA) targeting Myc tag at the carboxyl terminus of E2F1. Co-precipitated chromatin was eluted from complexes and purified by QIAquick PCR Purification Kit (QIAGEN, Valencia, CA, USA). The presence of *SIRT1 *and *APAF1 *promoter was analyzed by semi-quantitative PCR using 2 μl from 35 μl of DNA extraction and GoTaq DNA Polymerase (Promega, Madison, WI, USA). Primer sequences used were: Apaf-1 pro-F, 5'-GGAGACCCTAGGACGACAAG-3'; Apaf-1 pro-R, 5'-CAGTGAAGCAACGAGGATGC-3'. Primers specific for the *SIRT1 *promoter have been described previously [41]. PCR products were resolved on 2% agarose gel containing ethidium bromide.

### Quantitative real-time PCR

Total RNA was extracted using TriPure (Roche, Indianapolis, IN, USA). Total RNA (1 μg) was subjected to cDNA synthesis using an iScrip cDNA Synthesis Kit (Bio-Rad). Expression of target genes was examined using specific primers in combination with SsoFast EvaGreen Supermix using a CFX96 Real-Time PCR Detection System (Bio-Rad). Primers used for detecting E2F1 targets were: SIRT1-F, 5'-TGGCAAAGGAGCAGATTAGTAGG-3'; SIRT1-R, 5'-TCATCCTCCATGGGTTCTTCT-3'; Cyclin E1-F, 5'-GGTTAATGGAGGTGTGTGAAGTC-3'; Cyclin E1-R, 5'-CCATCTGTCACATACGCAAACT-3'; APAF1-F, 5'-TGACATTTCTCACGATGCTACC-3'; APAF1-R, 5'-ATTGTCATCTCCCGTTGCCA-3'; GAPDH-F, 5'-GTGGACCTGACCTGCCGTCT-3'; GAPDH-R, 5'-GGAGGAGTGGGTGTCGCTGT-3'. Primers used for determining transfection efficiency were: E2F1-F, 5'-GCTGAAGGTGCAGAAGCGGC-3'; E2F1-R, 5'-TCCTGCAGCTGTCGGAGGTC-3'; EGFP-F, 5'-CTACGGCGTGCAGTGCTTCA-3'; EGFP-R, 5'- CGCCCTCGAACTTCACCTCG-3'.

Relative expression levels of transcripts were normalized with glyceraldehyde 3-phosphate dehydrogenase (*GAPDH*) expression level.

### E2F1 over-expression

*E2F1 *plasmids were transiently transfected into MSTO-211H and NCI-H28 cells through the use of Effectene (QIAGEN) according to the manufacturer's instructions. Briefly, cells were plated at a density of 60% in a six-well plate. The next day, cells were transfected with 0.4 μg pcDNA6-E2F1, pcDNA6-E2F1/R166H or empty vector using Effectene. After a 48-hour transfection period, the cells were harvested for downstream assays. To determine transfection efficiency, 0.1 μg of pcDNA3-EGFP was co-transfected with 0.3 μg of *E2F1 *plasmids. Cells were collected for RNA and protein extraction after a 48-hour transfection. Expression of *EGFP *and *E2F1 *transcripts was assessed by real-time PCR.

### Western blot analysis

Cells were lysed in phosphate-buffered saline containing 1% triton-X100 in the presence of protease inhibitor (Roche, Indianapolis, IN, USA). Total protein extracts (20 μg) were separated using 8% SDS-PAGE, transferred to nitrocellulose membranes and probed with antibody specific for E2F1 (KH95; Santa Cruz Biotechnology, Santa Cruz, CA, USA) and β-actin (AC-15; Sigma).

### Degradation assay

MSTO-211H cells were transfected with 4 μg of wild-type E2F1 or E2F1-R166H in a 99-mm dish. After 24 hours, cells were harvested and split into a six-well plate. After 20 hours, cells were treated with RPMI containing 25 μg/ml cycloheximide (Sigma). Cells were collected at 30 minute time points and lysed in lysis buffer containing 1% triton-X100 and protease inhibitor. The E2F1 level was then determined by western blot.

### Proliferation assay

Transfected cells were seeded into a 96-well plate at a density of 2 × 10^3 ^cells after a 48-hour transfection period. Proliferation rates for cells over-expressing wild-type E2F1 and E2F1-R166H were assessed using the colorimetric 3-(4,5-dimethylthiazol-2yl)-5-(3-carboxymethoxyphenyl)-(4-sulfophenyl)-2H-tetrazoluim assay according to the manufacturer's protocol (MTS; Promega). The assay was performed in triplicate and repeated three times independently.

### Statistical analyses

Statistical analyses were performed with PASW Statistics 18.0 (IBM, Endicott, NY, USA). Differences between individual groups were analyzed using ANOVA followed by *post hoc *analysis. *P*-values of < 0.05 are considered statistically significant.

## Abbreviations

AP1: activator protein 1; BWA: Burrows-Wheeler Aligner; ChIP: chromatin immunoprecipitation; DP: E2F dimerization partner; E2F1: E2F transcription factor 1; EGFP: enhanced green fluorescent protein; GAPDH: glyceraldehyde 3-phosphate dehydrogenase; MEKK3: mitogen-activated protein kinase kinase kinase 3; MPM: malignant peritoneal mesothelioma; ORF: open reading frame; PPFIBP2: liprin beta 2; Rb: retinoblastoma protein 1; SNP: single nucleotide polymorphism; SNV: single nucleotide variant; TRAF7: TNF receptor-associated factor 7; WDPMP: well differentiated papillary mesothelioma of the peritoneum.

## Competing interests

The authors declare that they have no competing interests.

## Authors' contributions

PT, KCS and MT conceived of the study and participated in its design, coordination, and interpretation. BTT conceived of the study and participated in its design, coordination, and interpretation and critically revised the manuscript. WY drafted the manuscript and participated in the sequence alignment and design of the SNV discovery pipeline, carried out copy number analysis, conservation analysis and homology modeling of E2F1 and critically revised the manuscript. WCO carried out the over-expression study, ChIP study, and degradation assay, performed the statistical analysis, helped to draft the manuscript and critically revised the manuscript. SR and IC participated in the sequence alignment and design, and implementation and execution of the SNV discovery pipeline. CKO participated in the design and interpretation and carried out the initial isolation of mutant E2F1 from the tumor and cell line for downstream studies. GEA participated in the sequence alignment and helped with the interpretation of computational results. SSM, BW and KHL collected the patient samples, carried out the DNA extraction, established the cell line and performed the Sanger validations of candidate mutations. PMV participated in the interpretation of experimental data and provided additional insight into the mutation's functions. All authors have read and given approval of the version to be published.

## Supplementary Material

Additional file 1 **Sequencing coverage at *CDKN2A*, *RASSF1A *and *NF2***. Each graph shows the exons (brown box) and introns (brown line) as defined by ENSEMBL, the chromosome and chromosomal coordinates of the gene, the actual capture region as defined by Agilent SureSelect Human All Exon Kit v1.01 (gray box with green outlines or green lines if the capture region is very small relative to the distance between exons), and three plots showing sequencing depth versus chromosomal coordinates for the tumor, the normal sample and the cell line. Click here for file

Additional file 2 **Full candidate somatic mutation set with validation using Sanger sequencing**. Full data set containing computationally predicted somatic single nucleotide alterations with a quality by depth of at least three. The data set was also validated using Sanger sequencing. Click here for file

Additional file 3 **Sanger sequencing validation of *E2F1*, *PPFIBP2 *and *TRAF7 *for tumor, normal and cell line samples**. Heterozygous mutation (red arrow) on *E2F1*, *PPFIBP2*, and *TRAF7 *presented in the tumor and cell line compared to the normal sample. Click here for file

Additional file 4 **Relative expression of *E2F1 *wild type or *E2F1 *mutant after co-transfection with *EGFP *in MSTO-211H and NCI-H28 cells**. *E2F1 *levels were normalized to *EGFP *levels in each condition. Similar levels of transcripts of the R166H mutant and wild type *E2F1 *were observed. Click here for file

Additional file 5 **Schematic for detection of somatic single nucleotide variants in high-throughput sequencing data**. Flowchart describing computational detection of somatic single nucleotide variants in exome sequencing data. Click here for file
