# Supplementary figures and images for: First somatic mutation of E2F1 in a critical DNA binding residue discovered in well-differentiated papillary mesothelioma of the peritoneum
Source: Genome Biol. 2011 Sep 28;12(9):R96. doi: 10.1186/gb-2011-12-9-r96 (PMC3308059; doi:10.1186/gb-2011-12-9-r96)

## Slide 1
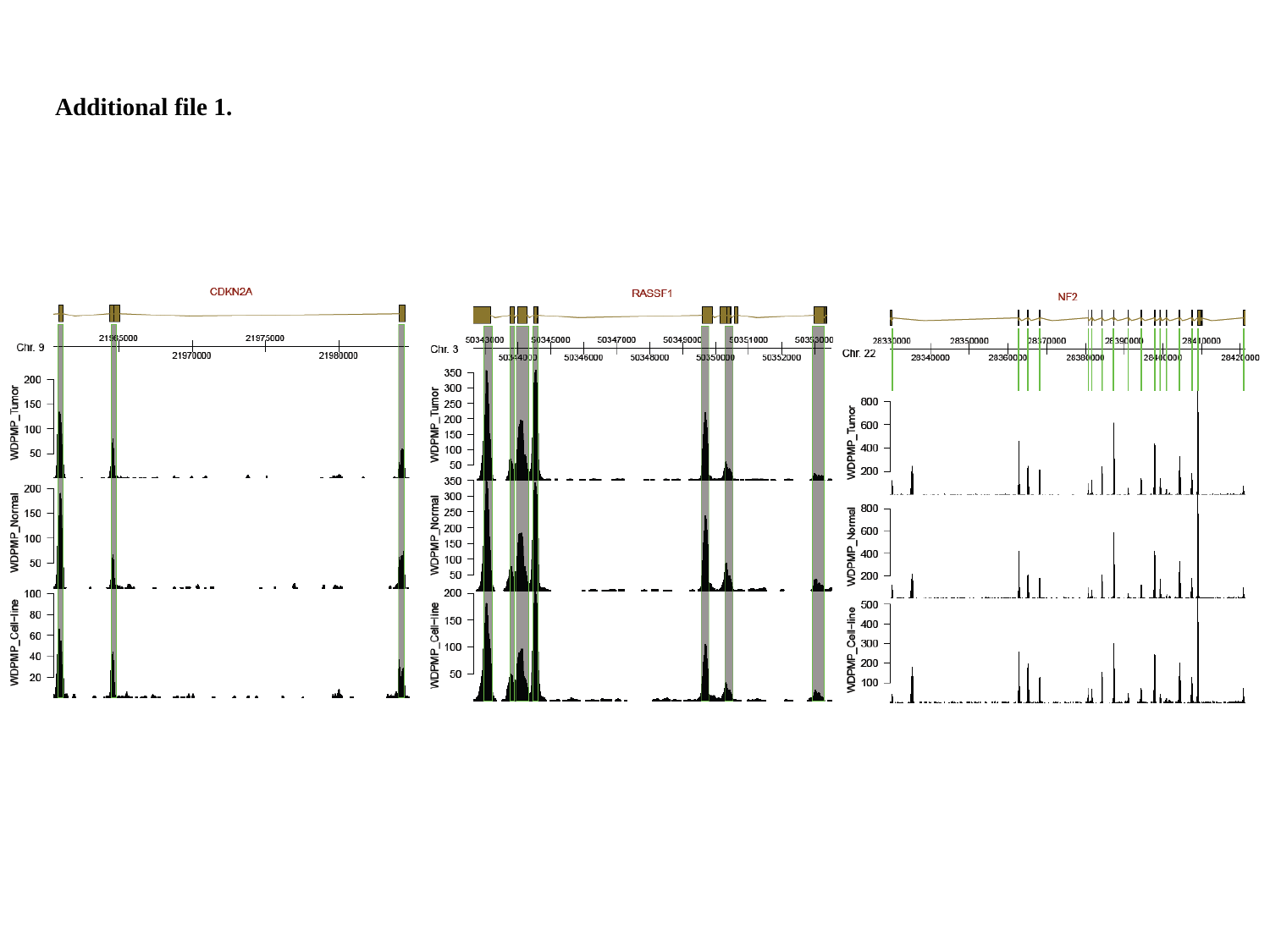

Additional file 1.

Supplement: Additional file 1 — Sequencing coverage at CDKN2A, RASSF1A and NF2. Each graph shows the exons (brown box) and introns (brown line) as defined by ENSEMBL, the chromosome and chromosomal coordinates of the gene, the actual capture region as defined by Agilent SureSelect Human All Exon Kit v1.01 (gray box with green outlines or green lines if the capture region is very small relative to the distance between exons), and three plots showing sequencing depth versus chromosomal coordinates for the tumor, the normal sample and the cell line. [file gb-2011-12-9-r96-S1.PPTX]

## Slide 1
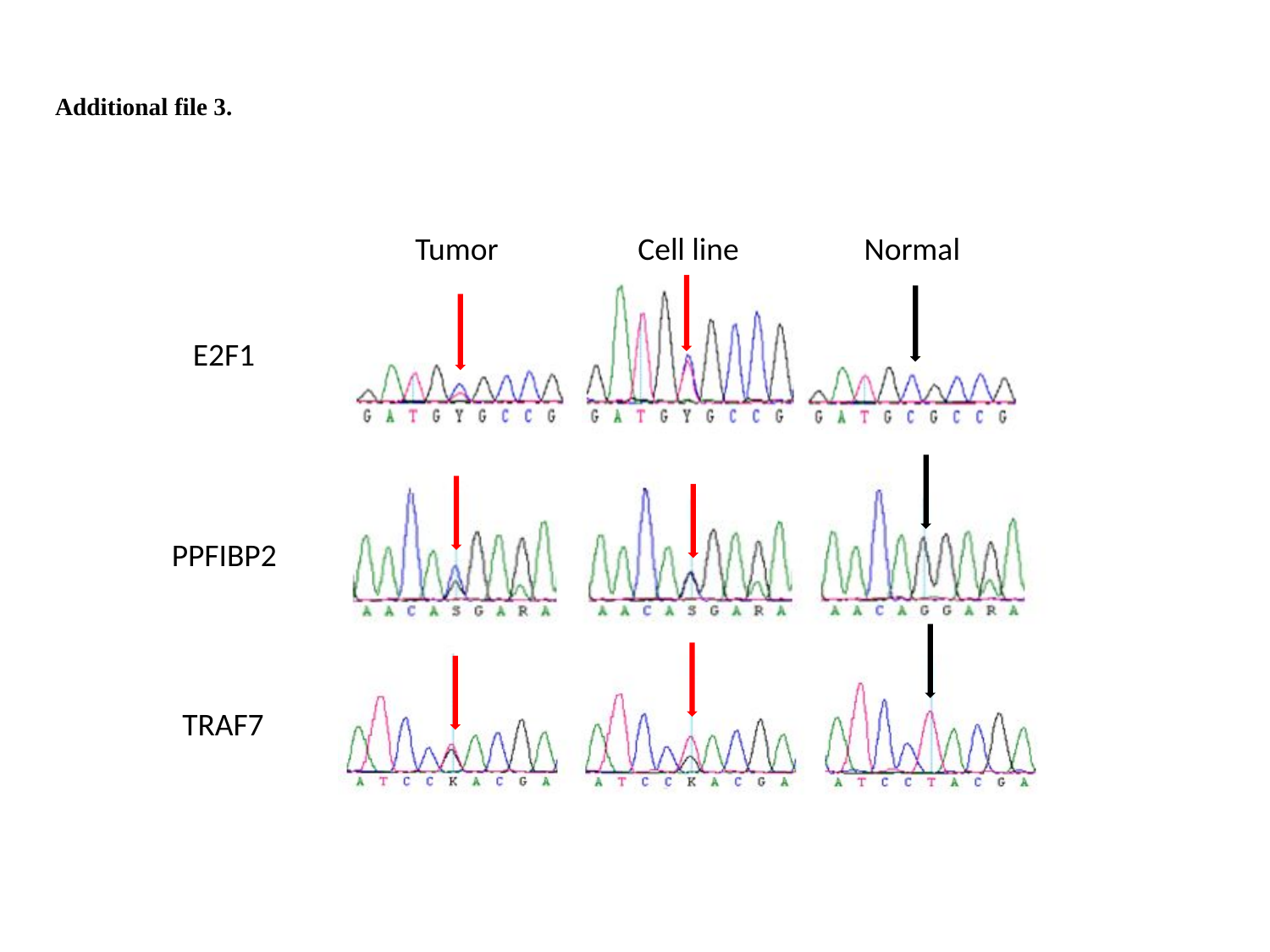

Additional file 3.
Tumor
Cell line
Normal
E2F1
PPFIBP2
TRAF7

Supplement: Additional file 3 — Sanger sequencing validation of E2F1, PPFIBP2 and TRAF7 for tumor, normal and cell line samples. Heterozygous mutation (red arrow) on E2F1, PPFIBP2, and TRAF7 presented in the tumor and cell line compared to the normal sample. [file gb-2011-12-9-r96-S3.PPTX]

## Slide 1
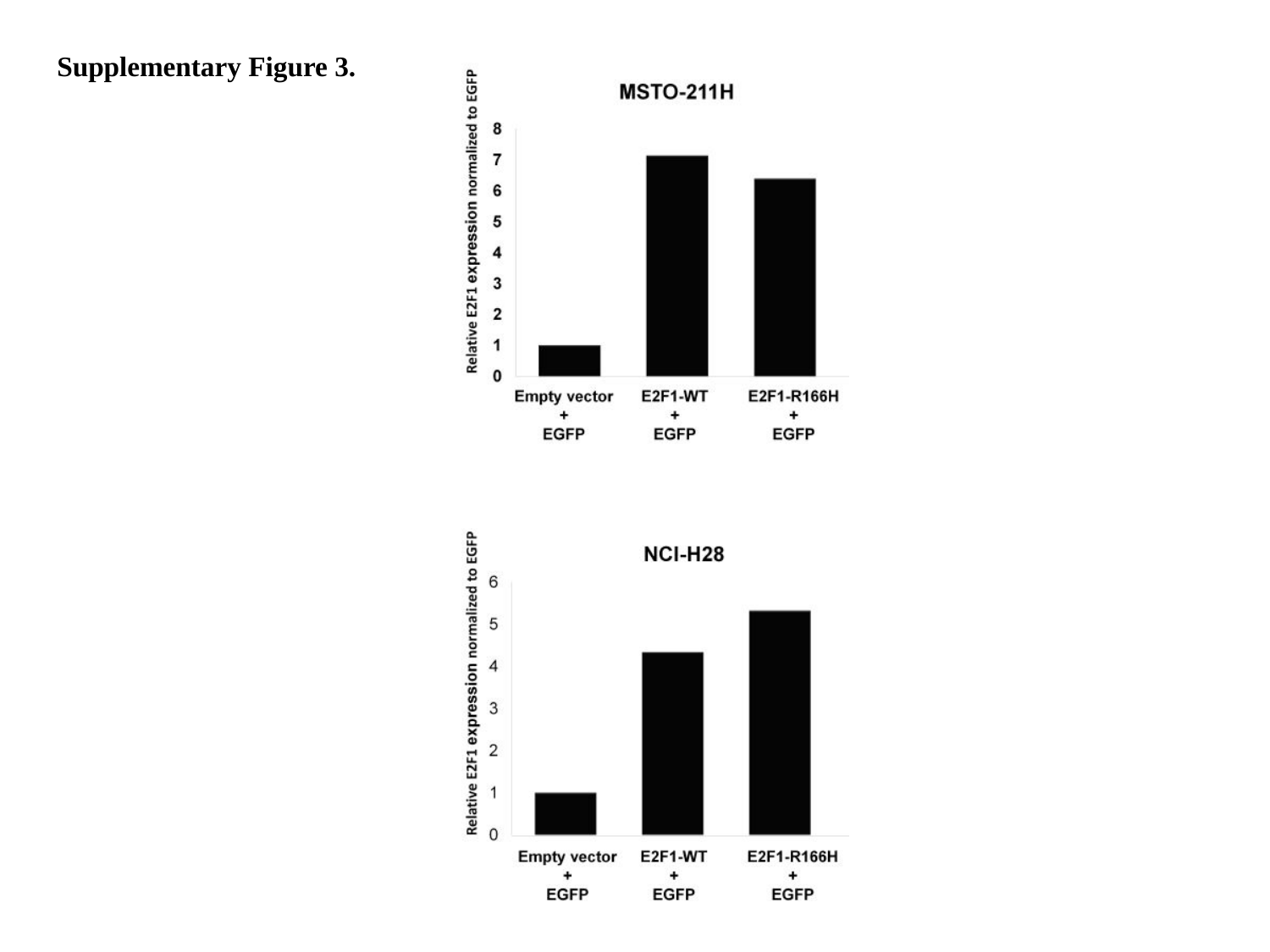

Supplementary Figure 3.

Supplement: Additional file 4 — Relative expression of E2F1 wild type or E2F1 mutant after co-transfection with EGFP in MSTO-211H and NCI-H28 cells. E2F1 levels were normalized to EGFP levels in each condition. Similar levels of transcripts of the R166H mutant and wild type E2F1 were observed. [file gb-2011-12-9-r96-S4.PPTX]
