# Supplementary material for: First somatic mutation of E2F1 in a critical DNA binding residue discovered in well-differentiated papillary mesothelioma of the peritoneum
Source: Genome Biol. 2011 Sep 28;12(9):R96. doi: 10.1186/gb-2011-12-9-r96 (PMC3308059; doi:10.1186/gb-2011-12-9-r96)
Supplement: Additional file 5 — Schematic for detection of somatic single nucleotide variants in high-throughput sequencing data. Flowchart describing computational detection of somatic single nucleotide variants in exome sequencing data. [file gb-2011-12-9-r96-S5.PPTX]

## Slide 1
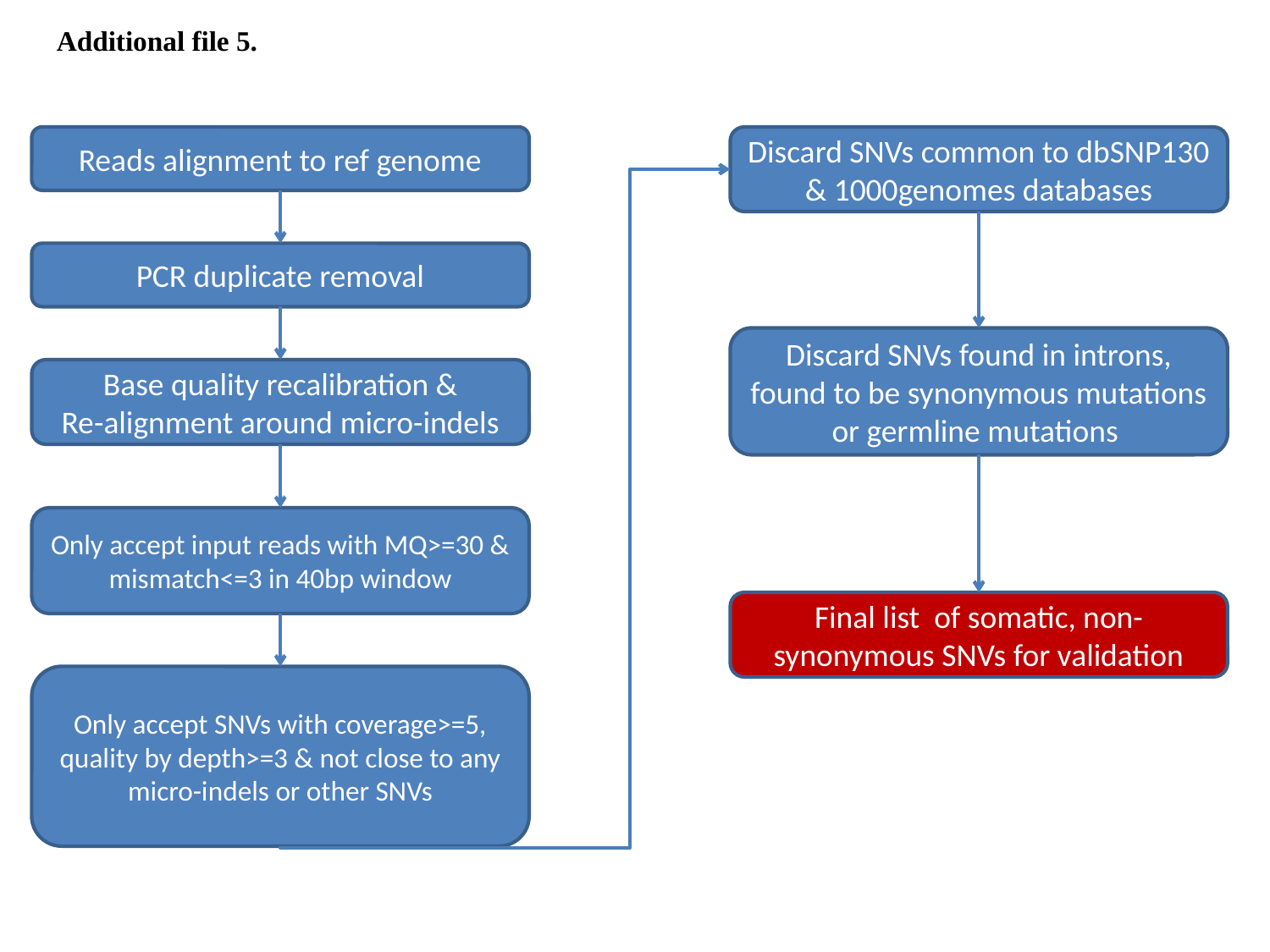

Additional file 5.
Reads alignment to ref genome
Discard SNVs common to dbSNP130 & 1000genomes databases
PCR duplicate removal
Discard SNVs found in introns, found to be synonymous mutations or germline mutations
Base quality recalibration &
Re-alignment around micro-indels
Only accept input reads with MQ>=30 & mismatch<=3 in 40bp window
Final list of somatic, non-synonymous SNVs for validation
Only accept SNVs with coverage>=5, quality by depth>=3 & not close to any micro-indels or other SNVs
